# Factors Associated with Preterm Birth and Low Birth Weight in Abu Dhabi, the United Arab Emirates

**DOI:** 10.3390/ijerph17041382

**Published:** 2020-02-21

**Authors:** Zainab Taha, Ahmed Ali Hassan, Ludmilla Wikkeling-Scott, Dimitrios Papandreou

**Affiliations:** 1 Department of Health Sciences, College of Natural and Health Sciences, Zayed University, Abu Dhabi 144534, UAE; ludmilla.scott@gmail.com (L.W.-S.); Dimitrios.Papandreou@zu.ac.ae (D.P.); 2 Taami for Agricultural and Animal Production, Khartoum, Sudan; aa801181@gmail.com

**Keywords:** preterm birth, low birth weight, caesarean section, maternal education, United Arab Emirates

## Abstract

Both preterm birth and low birth weight (LBW) represent major public health problems worldwide due to their association with the catastrophic effects of morbidity and mortality. Few data exist about such adverse pregnancy outcomes. The current study aimed to investigate the prevalence of and factors associated with preterm birth and LBW among mothers of children under two years in Abu Dhabi, United Arab Emirates. Data were collected in clinical and non-clinical settings across various geographical areas in Abu Dhabi. The data were analyzed using both descriptive and inferential statistics. A total of 1610 mother–child pairs were included in the current study. Preterm birth rate was 102 (6.3%) with a 95% confidence interval [CI] (6.1%, 6.5%) and the LBW rate was 151 (9.4%) with a 95% CI (9.3%, 9.5%). The mean (SD) of gestational age (GA) and birth weight at delivery was 39.1 (1.9) weeks and 3080.3 (518.6) grams, respectively. Factors that were positively associated with preterm birth were Arab mothers, maternal education level below secondary, caesarean section, and LBW. LBW was associated with female children, caesarean section (CS), first child order, and preterm birth. The current study highlighted the need for further interventional research to tackle these public health issues such as reducing the high CS rate and improving maternal education.

## 1. Introduction

According to the World Health Organization (WHO), every year an estimated 15 million babies are born preterm (<37 weeks of gestation) and this global trend is rising. Among 184 countries, the rate of preterm birth ranges from 5% to 18% of babies born [1]. Reports indicate a trend of preterm birth rate from 9.8% (8.3–10.9) in 2000 to 10.6% (9.0–12.0) in 2014. Of the estimated 14.84 million preterm births in 2014, the majority (81·1%) occurred in Asia and sub-Saharan Africa [2].

Preterm birth complications are considered the leading cause of death among children under five years of age and in 2015, they were responsible for approximately 1 million deaths [3]. A variety of factors including demographic and socioeconomic status have been reported to be associated with preterm birth such as maternal age, parity, previous preterm birth, multiple gestation, pregnancy induced hypertension, antepartum hemorrhage, prolonged pre-labor rupture of membranes, and urinary tract infections [4].

Another major health problem is Low Birth Weight (LBW). According to WHO, LBW is defined as a birth weight of less than 2500 g [5]. LBW may result from preterm birth, intrauterine growth restriction (IUGR), or both [6]. WHO has estimated that more than 20 million LBW infants are born annually [5]. These LBW infants are at an increased risk of several health problems such as growth retardation, infectious diseases, and developmental delay, which may occur during infancy, childhood, and ultimately, later stages of life [7]. Therefore, to protect infants and young children’s health, WHO has set a target of a 30% reduction in LBW by 2025 [5]. The reduction of LBW rates globally has also been considered as an important target of the United Nation’s Millennium Development Goal (MDG) for reducing child mortality [8].

A meta-analysis study identified several long-term negative outcomes associated with both preterm birth and LBW, such as lower educational qualifications, decreased rate of employment, and an increased rate of receipt of social benefits in adulthood [9].

According to the joint UNICEF and WHO study of global, regional, and country estimates of LBW in 2000, the incidence of LBW in the Middle East and the Gulf countries was reported as Oman (9%), Lebanon (6%), Syria (6%), Algeria (7%), Kuwait (7%), Libya (7%), Tunisia (7%), Bahrain (8%), Jordan (10%), Qatar (10%), Morocco (11%), Saudi Arabia (11%), Egypt (12%), and Yemen (32%) [10].

In Oman, study results confirm that high rates of consanguinity, premature births, number of increased pregnancies at an older maternal age, and changing lifestyles are some important factors related to the increasing rate of LBW [11]. Several studies have indicated a significant correlation between preterm birth and LBW [12,13,14]. Therefore, logic follows that these two adverse pregnancy outcomes should be investigated together and not merely separately. In Abu Dhabi, it was documented that LBW babies were 30.83 times more likely to require treatment in the neonatal intensive care unit in comparison to the babies of a normal birth weight [15]. The catastrophic effects of both preterm birth and LBW in terms of morbidity and mortality for both short and long term consequences, such as an increased rate of caesarean sections (CS), stillbirth, neonatal asphyxia, and mortality, were documented in the literature [9,16,17].

In the United Arab Emirates (UAE), data are limited regarding these adverse pregnancy outcomes, i.e., preterm birth and LBW, especially at the community level. Most available data for Abu Dhabi are not up to date and are mainly clinical in form [15].

The current study aimed to investigate the prevalence of and factors associated with preterm birth and low birth weight among mothers of children under two years in Abu Dhabi, United Arab Emirates.

## 2. Materials and Methods

### 2.1. Participants and Data Collection

This study’s sample is based on secondary data from an original sample obtained from mothers with at least one child under the age of two years. Participants for the original study included UAE nationals and non-nationals in the Emirate of Abu Dhabi, which represents 87% of the geographical landmass of UAE [18]. All data were collected between March and September of 2017 from the community and seven Ambulatory Maternal Child Health Centers. Mothers with young children attending the centers as well as from the community were approached by trained bilingual (Arabic and English) female research assistants who provided oral and written information about the study. Consenting mothers who met the inclusion criteria of having at least one singleton birth child under two years of age were interviewed by the research assistants using a structured questionnaire. The study was approved (ZU17_006_F) by the Research Ethics Committee at Zayed University UAE. In addition, another clearance was obtained from the Abu Dhabi Health Services Company. Informed consent was gained from all participants. Both confidentiality and privacy were maintained by excluding all personal identifiers during the period of data collection.

### 2.2. Study Instrument

A pre-tested questionnaire included family sociodemographic (e.g., age, nationality, education, occupation, family income, etc.) and child information (e.g., gestational age at delivery, birth weight, mode of delivery, child gender, etc.).

Birth outcomes (e.g., gestational age and birth weight) information was provided from the children’s health cards.

More details regarding the methodology of the primary data were described in the previous study [19].

### 2.3. Study Inclusion and Exclusion Criteria

From the total 1822 mother–child pairs, any data with completed variables were included in the analysis (*N* = 1610). The remaining 212 participants were excluded due to some missing data such as maternal education, paternal education, GA, mode of delivery, etc.

### 2.4. Statistical Analysis

The data were analyzed using Statistical Product and Service Solutions (IBM SPSS Statistics for Windows, Version 20.0. IBM Corp., Armonk, NY, USA). The data were analyzed using both descriptive and inferential statistics. Gestational age at delivery (term and preterm) and birth weight at delivery (normal birth weight and LBW) were analyzed as the dependent variables, independently. Term babies were coded as (0) and preterm babies were coded as (1). Normal weight birth was coded as (0) and LBW as (1). Other variables such as sociodemographic characteristic (e.g., age, nationality, parent education, maternal occupation, etc.) and child information (e.g., child gender, mode of delivery, etc.) were considered the independent variables.

*T*-test and Chi-square tests were applied to analyze the continuous and categorical data, respectively. Furthermore, significant continuous variables, for example, child order (1st order and >1st order) were categorized for analysis. Variables that were found to be statistically significant (*p*-value < 0.05) in the bivariate analysis were then further analyzed using multivariable logistic analysis for each dependent variable (preterm and LBW). To ensure the robustness of the logistic models, the insignificant variables in the bivariate analysis were also added to the models and stepwise model selection methods were applied. Finally, odds Ratio [OR] and 95% Confidence Interval [CI] were calculated with a significance level of *p*-value < 0.05.

### 2.5. Definitions

Gestational age (GA) was defined as a measure of the age of a pregnancy in weeks, which is taken from the beginning of the woman’s last menstrual period. Term birth was defined as the birth of a baby at ≥37 weeks GA. Preterm birth was defined as the birth of a baby at <37 weeks GA. Normal birth weight was defined as the weight of a baby immediately after delivery (≥2500 g). Low birth weight was defined as the weight of a baby immediately after delivery (<2500 g). Arab nationality was defined as those who self-identified themselves as Emirati or another Arab origin. Non-Arab nationality was defined as those who self-identified themselves as Asian or any other non-Arab origin. Family income was defined based on the mother’s answer to the following question, “Considering your monthly family income, how would you rate your and your family’s overall financial well-being?” <good or ≥good.

## 3. Results

A total of 1610 mother–child pairs were included in the study from the original sample (*N* = 1822). The mean (standard deviation) [SD] of maternal age and children’s age were 30.1 (5.1) years and 8.1 (5.9) months, respectively.

Table 1 describes the characteristics of the mothers based on the gestational age of the study children.

Among the LBW (151), 70 (46.3%) reportedly delivered vaginally, 51 (33.8%) by planned CS, and 30 (19.9%) by emergency CS.

In multivariable stepwise logistic regression analysis (Table 2), factors that were positively associated with preterm birth were Arab mothers (adjusted odds ratio [AOR] 2.02, 95% CI 1.19, 3.43), maternal education level below secondary (AOR 4.38, 95% CI1.95, 9.81), CS (AOR 2.35, 95% CI 1.48, 3.73), and LBW (AOR 17.62, 95% CI 11.05, 28.10).

Table 3 describes the characteristics of mothers based on the birth weight of the study children.

In an additional multivariable stepwise logistic regression analysis (Table 4), factors that were positively associated with LBW were female child (AOR 2.08, 95% CI 1.41, 3.08), CS (AOR 2.29, 95% CI 1.57, 3.35), first child order (AOR 1.98, 95% CI 1.35, 2.89), and preterm birth (AOR 17.64, 95% CI 11.03, 28.21).

## 4. Discussion

The main findings of the current study are the estimation of rates for both preterm birth 6.3% and LBW 9.4% and the identification of the main risk factors associated with preterm birth and LBW in Abu Dhabi, UAE.

The rate of preterm birth (6.3%) was slightly higher in comparison to the previous rate reported in Abu Dhabi (6%) [19], but was similar to Saudi Arabia (6.5%) [2] and lower than those found in Oman, 9.7% [14], and Kenya, 18.3% [4].

A similar LBW rate of 9.4% was reported in Iran [20]. However, the rate of LBW in the current study is higher than what was previously reported by UNICEF, namely, the country estimates were 6.1% [21] and for Abu Dhabi, in particular, 8.8% [15]. However, the current LBW rate (9.4%) is lower than previously reported and as compared to neighboring countries such as Yemen, 18% [22], Oman, 13.7% [14], and the African continent, as seen in Sudan, 12.5% [23], Ethiopia, 10.4% [13], and Nigeria, 16% [24] or South East Asia as seen in Pakistan, 10.04% [16]. The differences in rates could be explained by the nature of the studies, for instance, delivery at tertiary hospitals [23,24] may be associated with high preterm births due to dealing with complicated pregnancies, such as preterm birth, unlike the current study, which was community-based.

The current study showed that preterm birth and LBW were significantly associated with each other, i.e., a preterm baby is almost 18 times more at risk of being LBW and vice versa. Consistent with the present results, a plethora of studies have documented this significant association [16,25,26] which highlights the importance of intervention programs that aim at reducing both outcomes.

This study revealed that Arab mothers were twice as likely to deliver a preterm baby compared to non-Arab mothers. In line with the current results, several studies showed similar findings [27,28]. Some of the important pregnancy outcomes, such as preterm birth and LBW, were found to vary by nationality [28]. Such differences among nationalities require more investigation, especially in a country like the UAE with multiple nationalities [18].

In the present study, maternal education was found to be associated only with preterm birth, and mothers who had below secondary level education had a four times higher risk of delivering a preterm baby. This is in line with previous observations in different countries including Gulf countries [29,30]. The literature has revealed significant associations between low maternal education and the risk of poor neonatal health outcomes [23,31]. For instance, in Italy, a population-based study revealed that low maternal education was a risk factor for both preterm birth and LBW [31].

Female gender was found to be at double the risk of having LBW (AOR 2.08, 95% CI 1.41, 3.08) than their counterparts. The present results are in agreement with the previous studies, including the neighboring country Oman [12,14].

There are several explanations for the difference in LBW by baby’s gender and it should be noted that male babies usually weigh more than their female counterparts, which may serve as a protective factor [32]. In addition, there is an increase in mortality rates which are associated with LBW male gender counterparts as the gender differences in infants’ mortality were observed in the previous studies [33], especially among male infants born between 24 and 26 weeks [34]. For example, systematic review and meta-analysis of more than 30 million births showed an elevated risk of stillbirth in males by about 10% [33]. Therefore, to have an accurate estimation of LBW and its associated factors, future research needs to take into account perinatal mortality and its association with gender.

A baby who is delivered by CS was almost 2.5 times more at risk of being preterm and LBW. Similar to the current results, previous studies found CS to be associated with both preterm birth [35] and LBW [17]. However, the preterm births were not classified into subtypes i.e., spontaneous versus indicated [36], and more than half of the preterm birth babies were delivered via CS (55.9%). This is in line with the trend of increasing indicated preterm deliveries over spontaneous ones [37].

Unfortunately, various studies have documented an epidemic of CS which has been reported to have adverse pregnancy outcomes, such as preterm birth and LBW [35,36,37,38]. Among preterm births and LBW that were delivered by CS, more than half of those deliveries were planned. This might be due to the abuse of planned CS as it was observed to be wrongfully associated with term LBW, especially in private hospitals in Brazil [39]. Adding to that, in the present study, 62.3% of the LBW were term LBW. Therefore, WHO’s recommendations [1] need to be followed, namely, the induction or caesarean birth should not be planned before 39 completed weeks unless medically indicated for the benefit of the mother, the fetus, or both. In addition, the effect of CS is not only confined to the index birth, but it was identified as a risk factor for preterm birth in subsequent births as compared to vaginal delivery [40,41].

There are some controversial results around the association between LBW and CS. While some researchers reported CS as a protective factor [27], others in the nearby country reported this method of delivery as a risk factor for LBW [20], similar to the present findings. For example, Hailu and Kebede [27] reported that Cesarean delivery (AOR 0.415, 95% CI 0.183, 0.941) had a preventive effect of LBW. This could be explained by the CS rates. When the rate is within the WHO recommended range, it will be protective against LBW; otherwise it will be a risk. Therefore, all efforts should be directed to reduce the high rate and to identify the possible indications of CS in the studied area. This study can be considered as a call to action to reduce the high rates of caesarean section in the UAE and across the globe.

The risk for LBW nearly doubled among first child order (AOR 1.98, 95% CI 1.35, 2.89). This finding was supported by various other studies in Sudan [23], Pakistan [16], China [41], and India [42].

Both not having previous children and caesarean births were associated with preterm birth and LBW [43], however in the current study, first child order was not associated with preterm birth. The association between first child order and LBW may be due to the increased rate of CS among primipara mothers [44].

Unlike the current study, previous research found preterm birth and LBW to be associated with other factors as well—for example, LBW with maternal age [12,45] and maternal BMI [45,46] and preterm birth with maternal age [4,14] and maternal BMI [14,46,47].

The discrepancies in factors associated with preterm and LBW as reported in the literature could be explained by the interrelation of various factors. For example, in Finland, Goisiset al. [48] reported that advanced maternal age is not independently associated with the risk of preterm birth or LBW among mothers who have had at least two live births. This should encourage researchers to investigate their local area and to develop solutions based on the local findings.

This study revealed valuable information from a large sample of the community, which can be used by healthcare planners to improve pregnancy outcomes. However, some limitations need to be considered while analyzing the current results and need to be covered in future studies, aiming to give a better description for these important public health issues among the UAE populations. First, this study did not include morbidity information among the study participants (both mothers and children) and mortality information among LBW children who were not included in the sample (i.e., hospitalized infants and subsequent deaths). This could be of importance as the literature documented a high rate of mortality among this vulnerable group [23,49], i.e., this may cause underestimation of the LBW rate. Second, factors related to maternal health that were found to be associated with adverse pregnancy outcomes, such as anemia [49,50] and periodontal diseases [51,52], were not included. Third, the missing data from the original study might bias the current results.

## 5. Conclusions

The rates of preterm birth and LBW were found to be high in our study. In addition, preterm birth was found to be strongly positively associated with LBW. The current study highlights the need for further interventional research to tackle these public health issues, such as reducing the high CS rate and improving maternal education.

## Figures and Tables

**Table 1:** Characteristics of the mothers with children below two years of age in Abu Dhabi, the UAE, from March to September 2017 based on gestational age at delivery.

Variables		Gestational Age						
		Total (*N* = 1610)		Term (*n* = 1508)		Preterm (GA < 37 weeks (*n* = 102)		
		Mean (SD)		Mean (SD)		Mean (SD)		*p*-Value
Maternal age, years		30.1 (5.1)		30.1 (5.1)		30.4 (5.6)		0.578
Child order		2.2 (1.2)		2.2 (1.2)		3.3 (1.3)		0.393
Birth weight, grams		3080.3 (518.6)		3127.3 (473.0)		2385.6 (652.6)		<0.001
Maternal pre-pregnancy BMI (kg/m^2^)		23.9 (3.9)		23.9 (3.7)		24.0 (5.5)		0.824
		*N*	%	*N*	%	*N*	%	*p*-value
Child gender	Male	790	49.1	743	49.3	47	46.1	0.533
	Female	820	50.9	765	50.7	55	53.9	
Nationality	Arab	1049	65.2	970	64.3	79	77.5	0.007
	Non-Arab	561	34.8	538	35.7	23	22.5	
Marital status	Married	1588	98.6	1489	98.7	99	97.1	0.157
	Divorced/Single	22	1.4	19	1.3	3	2.9	
Mode of delivery	Vaginal	1121	69.6	1076	71.4	45	44.1	<0.001
	CS	489	30.4	432	28.6	57	55.9	
Maternal education	<Secondary level	64	4.0	54	3.6	10	9.8	0.002
	≥Secondary level	1546	96.0	1454	96.4	92	90.2	
Paternal education	<Secondary level	31	1.9	27	1.8	4	3.9	0.130
	≥Secondary level	1579	98.1	1481	98.2	98	96.1	
Maternal occupation	Housewife	996	62.2	934	62.3	62	60.8	0.729
	Employed	606	37.8	566	37.7	40	39.2	
Family income	<Good	102	6.4	96	6.4	6	5.9	0.856
	≥Good	1504	93.6	1408	93.6	96	94.1	
Birth weight	Normal birth weight (≥2500 g)	1459	90.6	1414	93.8	45	44.1	<0.001
	Low birth weight (<2500 g)	151	9.4	94	6.2	57	55.9	

**Table 2:** Multivariable stepwise logistic regression analyses of factors associated with preterm birth among mothers with children below two years of age in Abu Dhabi, the UAE, from March to September 2017.

Variable		Crude Odds Ratio (95% CI)	Adjusted Odds Ratio (95% CI)	*p*-Value
Nationality	Arab versus Non-Arab	1.91 (1.18, 3.06)	2.02 (1.19, 3.43)	0.009
Maternal education	< Secondary versus ≥ Secondary	2.93 (1.44, 5.93)	4.38 (1.95, 9.81)	<0.001
Mode of delivery	Caesarean section versus Vaginal	3.16 (2.10, 4.74)	2.35 (1.48, 3.73)	<0.001
Birth weight	Low birth weight (<2500 g) versus Normal birth weight (≥2500 g)	19.05 (12.23, 29.68)	17.62 (11.05, 28.10)	<0.001

**Table 3:** Characteristics of mothers with children below two years of age in Abu Dhabi, the UAE, from March to September 2017 based on birth weight at delivery.

Variable		Birth Weight						
		Total (*N* = 1610)		Normal (*n* = 1459)		LBW (*n* = 151)		
		Mean (SD)		Mean (SD)		Mean (SD)		*p*-Value
Maternal age (years)		30.1 (5.1)		30.0 (5.2)		30.6 (5.0)		0.252
Child order		2.2 (1.2)		2.2 (1.2)		2.0 (1.2)		0.020
Gestational age, weeks		39.1 (1.9)		39.4 (1.6)		37.0 (3.1)		<0.001
Maternal pre-pregnancy BMI (kg/m^2^)		23.9 (3.9)		23.9 (3.9)		23.5 (3.8)		0.252
		*N*	%	*N*	%	*N*	%	*p*-Value
Child gender	Male	790	49.1	737	50.5	53	35.1	<0.001
	Female	820	50.9	722	49.5	98	64.9	
Nationality	Arab	1049	65.2	949	65.0	100	66.2	0.772
	Non-Arab	561	34.8	510	35.0	51	33.8	
Marital status	Married	1588	98.6	1439	98.6	149	98.7	0.963
	Divorced/Single	22	1.4	20	1.4	2	1.3	
Mode of delivery	Vaginal delivery	1121	69.6	1051	72.0	70	46.4	<0.001
	Caesarean section	489	30.4	408	28.0	81	53.6	
Maternal education	<Secondary level	64	4.0	59	4.0	5	3.3	0.661
	≥Secondary level	1546	96.0	1400	96.0	146	96.7	
Paternal education	<Secondary level	31	1.9	29	2.0	2	1.3	0.572
	≥Secondary level	1579	98.1	1430	98.0	149	98.7	
Maternal occupation	Housewife	996	62.2	891	61.4	105	69.5	0.096
	Employed	606	37.8	560	38.6	46	30.5	
Family income	<good	102	6.4	97	6.7	5	3.3	0.222
	≥good	1504	93.6	1358	93.3	146	96.7	
Child order	1st order	582	36.1	509	34.9	73	48.3	0.001
	>1st order	1028	63.9	950	65.1	78	51.7	
Gestational age	Term (GA ≥ 37 weeks)	1508	93.7	1414	96.9	94	62.3	<0.001
	Preterm (<37 weeks)	102	6.3	45	3.1	57	37.7	

**Table 4:** Multivariable stepwise logistic regression analyses of factors associated with low birth weight among mothers with children below two years of age in Abu Dhabi, UAE, from March to September 2017.

Variable		Crude Odds Ratio (95% CI)	Adjusted Odds Ratio (95% CI)	*p*-Value
Child gender	Female versus Male	1.89 (1.33, 2.68)	2.08 (1.41, 3.08)	<0.001
Mode of delivery	Caesarean section versus Vaginal	3.0 (2.12, 4.19)	2.29 (1.57, 3.35)	<0.001
Child order	1st order versus >1st order	1.75 (1.25, 2.45)	1.98 (1.35, 2.89)	<0.001
Gestational age	Preterm (<37 weeks) versus Term (GA ≥37 weeks)	19.05 (12.23, 29.68)	17.64 (11.03, 28.21)	<0.001
